# Supplementary material for: Multi-omics reveal neuroprotection of Acer truncatum Bunge Seed extract on hypoxic-ischemia encephalopathy rats under high-altitude
Source: Commun Biol. 2023 Oct 2;6:1001. doi: 10.1038/s42003-023-05341-9 (PMC10545756; doi:10.1038/s42003-023-05341-9)
Supplement: Supplementary file 2 — Supplementary materials [file 42003_2023_5341_MOESM2_ESM.pdf]

Supplementary materials

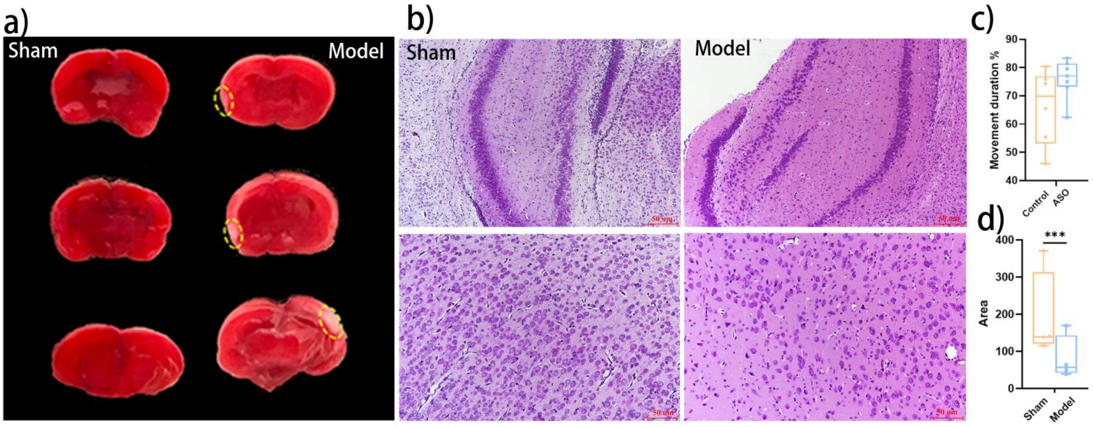

Supplementary Figure 1. a) TTC stain of brain tissues. **b)** HE staining showed histological changes in each group, and all three replicates had similar staining results. **c)** Representative the key indicators of open field test, including movement duration. **d)** Quantitative analysis of HE staining. */**/*** indicates *P*-value < 0.05 / < 0.01 / < 0.001.

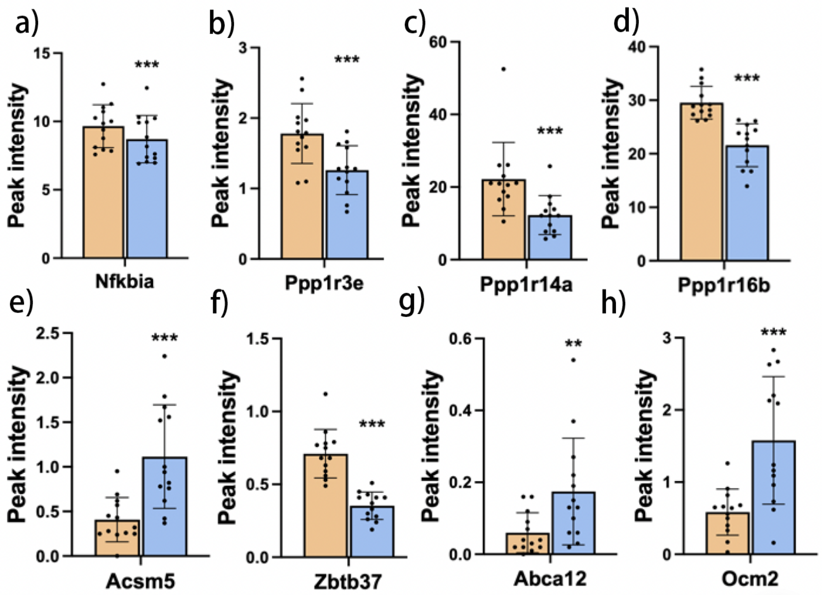

Supplementary Figure 2. a-h) Potential genes involved in each pathway of the mechanism. */**/*** indicate *P*-value < 0.05 / < 0.01 / < 0.001.
