# Supplementary material for: Multi-omics reveal neuroprotection of Acer truncatum Bunge Seed extract on hypoxic-ischemia encephalopathy rats under high-altitude
Source: Commun Biol. 2023 Oct 2;6:1001. doi: 10.1038/s42003-023-05341-9 (PMC10545756; doi:10.1038/s42003-023-05341-9)
Supplement: Supplementary file 3 — Reporting Summary [file 42003_2023_5341_MOESM3_ESM.pdf]

nature portfolio

Corresponding author(s): Jiujun Li

Last updated by author(s): Sep 3, 2023

Reporting Summary

Nature Portfolio wishes to improve the reproducibility of the work that we publish. This form provides structure for consistency and transparency in reporting. For further information on Nature Portfolio policies, see our Editorial Policies and the Editorial Policy Checklist.

Statistics

For all statistical analyses, confirm that the following items are present in the figure legend, table legend, main text, or Methods section.

n/a	Confirmed	
☐	☒	The exact sample size (*n*) for each experimental group/condition, given as a discrete number and unit of measurement
☐	☒	A statement on whether measurements were taken from distinct samples or whether the same sample was measured repeatedly
☐	☒	The statistical test(s) used AND whether they are one- or two-sided<br>*Only common tests should be described solely by name; describe more complex techniques in the Methods section.*
☒	☐	A description of all covariates tested
☐	☒	A description of any assumptions or corrections, such as tests of normality and adjustment for multiple comparisons
☐	☒	A full description of the statistical parameters including central tendency (e.g. means) or other basic estimates (e.g. regression coefficient) AND variation (e.g. standard deviation) or associated estimates of uncertainty (e.g. confidence intervals)
☐	☒	For null hypothesis testing, the test statistic (e.g. *F*, *t*, *r*) with confidence intervals, effect sizes, degrees of freedom and *P* value noted<br>*Give P values as exact values whenever suitable.*
☒	☐	For Bayesian analysis, information on the choice of priors and Markov chain Monte Carlo settings
☐	☒	For hierarchical and complex designs, identification of the appropriate level for tests and full reporting of outcomes
☒	☐	Estimates of effect sizes (e.g. Cohen's *d*, Pearson's *r*), indicating how they were calculated

Our web collection on statistics for biologists contains articles on many of the points above.

Software and code

Policy information about availability of computer code

Data collection	Aligning RNA-sequencing reads to the murine reference genome (https://ftp.ensembl.org/pub/release-101/fasta/-mus musculus/dna/) was performed using HISAT2 (https://daehwankimlab.github.io/hisat2/,version:hisat2-2.0.4). The mapped reads were assembled using StringTie (https://ccb.jhu.edu/software/stringtie/,version:stringtie-1.3.4). In the following step, all transcriptomes were merged using gffcompare software (https://ccb.jhu.edu/software/stringtie/gffcompare.shtml,version:gffcompare-0.9.8.) to reconstruct a comprehensive transcriptome. Following the generation of the transcriptome, StringTie and Ballgown (https://www.bioconductor.org/packages/release/bioc/html/ballgown.html) were used to estimate the expression levels of all transcripts. Metabolic changes in Plasma extract were analyzed by using the UPLC-Q-TOF MS system and the equipped software Progenesis QI (Waters).
Data analysis	Differentially expressed genes (DEGs) were identified by at least 2 fold changes and Q-value < 0.05 (DESeq2 R package, https://www.bioconductor.org/packages/release/bioc/html/-DESeq2.html). An analysis of gene differential expression was performed using DESeq2 software between two groups (and by edgeR between two samples). All statistical analyses were performed using R version 3.6.3, and P < 0.05 was considered statistically significant. Packages of “ropls” and “muma” were used for OPLS-DA analysis. Packages of “corrplot”, “corrgram”, “pheatmap”, “dendextend” and “nVennR” were used for visualization of correlations, heatmaps, cluster, and venn analyses. Customizing structural equation modelling plots was carried out by using "semptools" package.

For manuscripts utilizing custom algorithms or software that are central to the research but not yet described in published literature, software must be made available to editors and reviewers. We strongly encourage code deposition in a community repository (e.g. GitHub). See the Nature Portfolio guidelines for submitting code & software for further information.

Data

Policy information about availability of data

All manuscripts must include a data availability statement. This statement should provide the following information, where applicable:

- Accession codes, unique identifiers, or web links for publicly available datasets
- A description of any restrictions on data availability
- For clinical datasets or third party data, please ensure that the statement adheres to our policy

Availability of data and material: The corresponding author had full access to all the data in the study and take responsibility for the integrity of the data and the accuracy of the data analysis. The transcriptome data are stored in the SRA database, Accession to cite for these SRA data: PRJNA909348. Temporary Submission ID: SUB12485094. SRA records will be accessible with the following link after the indicated release date: https://www.ncbi.nlm.nih.gov/sra/PRJNA909348. The relevant metabolomics raw data and master alignment ion feature tables generated for this study have been deposited in the Metabolomics Workbench (datatrack_id:3673 study_id:ST002483). https://www.metabolomicsworkbench.org where it has been assigned Study ID ST002483. The data can be accessed directly via its Project DOI: http://dx.doi.org/10.21228/M87H9N This work is supported by NIH grant U2C-DK119886 and OT2-OD030544 grants. Please acknowledge them in your publications. Regards, NIH Common Fund's National Metabolomics Data Repository (NMDR).

Research involving human participants, their data, or biological material

Policy information about studies with human participants or human data. See also policy information about sex, gender (identity/presentation), and sexual orientation and race, ethnicity and racism.

Reporting on sex and gender	N/A
Reporting on race, ethnicity, or other socially relevant groupings	N/A
Population characteristics	N/A
Recruitment	N/A
Ethics oversight	N/A

Note that full information on the approval of the study protocol must also be provided in the manuscript.

Field-specific reporting

Please select the one below that is the best fit for your research. If you are not sure, read the appropriate sections before making your selection.

☒ Life sciences ☐ Behavioural & social sciences ☐ Ecological, evolutionary & environmental sciences

For a reference copy of the document with all sections, see nature.com/documents/nr-reporting-summary-flat.pdf

Life sciences study design

All studies must disclose on these points even when the disclosure is negative.

Sample size	The number of HIE rats were first determined by referring to transcriptomics and lipidomics articles on hypoxic-ischemia studies. Besides, based on our previous study, the power analysis was performed for differences in essential fatty acids to determine the number of HIE rats, using sig.level = 0.05, power = 0.8 and alternative = two.sided.
Data exclusions	Thirteen rats were used for the transcriptome study, and eight rats were used in the lipidomics study. Both control and ASO groups were the same. Behavioral studies assigned seven animals to each group, with one accidental death excluded from the control group.
Replication	All attempts at replication were successful.
Randomization	HIE rats were numbered, and then a set of random numbers were generated to group the corresponding rats.
Blinding	The investigators were blinded to group allocation during data collection and analysis.

Reporting for specific materials, systems and methods

We require information from authors about some types of materials, experimental systems and methods used in many studies. Here, indicate whether each material, system or method listed is relevant to your study. If you are not sure if a list item applies to your research, read the appropriate section before selecting a response.

Materials & experimental systems

n/a	Involved in the study
☒	☐ Antibodies
☒	☐ Eukaryotic cell lines
☒	☐ Palaeontology and archaeology
☐	☒ Animals and other organisms
☒	☐ Clinical data
☒	☐ Dual use research of concern
☒	☐ Plants

Methods

n/a	Involved in the study
☒	☐ ChIP-seq
☒	☐ Flow cytometry
☒	☐ MRI-based neuroimaging

Animals and other research organisms

Policy information about studies involving animals; ARRIVE guidelines recommended for reporting animal research, and Sex and Gender in Research

Laboratory animals	All animal experiments were performed according to protocols approved by the Scientific Research Ethics Committee of Shengjing Hospital affiliated with China Medical University. Sprague-Dawley pregnant rats at gestation days 18-21 were purchased from Liaoning Changsheng Biotechnology Co. Ltd (Permit number: SCXK Liaoning 2020-0002). All rats were housed in a temperature-controlled room (22-26?) under 12 h light and dark cycles, with free food and water throughout the study.
Wild animals	The study did not involve wild animals.
Reporting on sex	All postnatal 7-day-old rats of both genders were anesthetized with ether inhalation. In neonatal hypoxia and ischemia research, gender is not regarded as an independent variable. All rats were randomly assigned and there were no age or gender differences among them.
Field-collected samples	The study did not involve samples collected from the field.
Ethics oversight	All experiments performed in this study were following the Guide for the Care and Use of Laboratory Animals and were approved by the Ethics Committee of Medical ethics committee of Shengjing Hospital of China Medical University (No. P2020PS661K).

Note that full information on the approval of the study protocol must also be provided in the manuscript.
